# Supplementary material for: Causal Relationship between Aging and Anorexia Nervosa: A White-Matter-Microstructure-Mediated Mendelian Randomization Analysis
Source: Biomedicines. 2024 Aug 16;12(8):1874. doi: 10.3390/biomedicines12081874 (PMC11351342; doi:10.3390/biomedicines12081874)
Supplement: Supplementary file 1 [file biomedicines-12-01874-s001.zip › Supplementary Figure/Figure S4/Singe SNP plot of MR analysis for TL to ubm-b-1745.pdf]

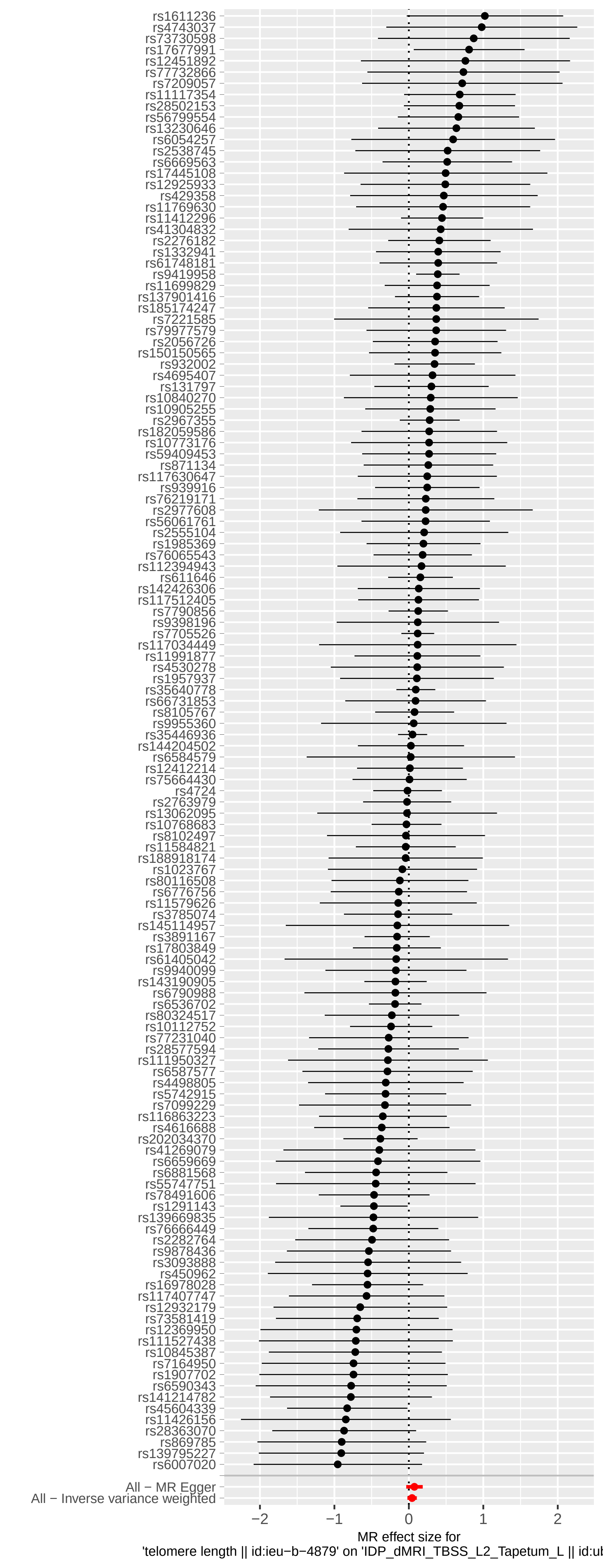
rs1611236
rs4743037
rs73730598
rs17677991
rs12451892
rs77732866
rs7209057
rs11117354
rs28502153
rs56799554
rs13230646
rs6054257
rs2538745
rs6669563
rs17445108
rs12925933
rs429358
rs11769630
rs11412296
rs41304832
rs2276182
rs1332941
rs61748181
rs9419958
rs11699829
rs137901416
rs185174247
rs7221585
rs79977579
rs2056726
rs150150565
rs932002
rs4695407
rs131797
rs10840270
rs10905255
rs2967355
rs182059586
rs10773176
rs59409453
rs871134
rs117630647
rs939916
rs76219171
rs2977608
rs56061761
rs2555104
rs1985369
rs76065543
rs112394943
rs611646
rs142426306
rs117512405
rs7790856
rs9398196
rs7705526
rs117034449
rs11991877
rs4530278
rs1957937
rs35640778
rs66731853
rs8105767
rs9955360
rs35446936
rs144204502
rs6584579
rs12412214
rs75664430
rs4724
rs2763979
rs13062095
rs10768683
rs8102497
rs11584821
rs188918174
rs1023767
rs80116508
rs6776756
rs11579626
rs3785074
rs145114957
rs3891167
rs17803849
rs61405042
rs9940099
rs143190905
rs6790988
rs6536702
rs80324517
rs10112752
rs77231040
rs28577594
rs111950327
rs6587577
rs4498805
rs5742915
rs7099229
rs116863223
rs4616688
rs202034370
rs41269079
rs6659669
rs6881568
rs55747751
rs78491606
rs1291143
rs139669835
rs76666449
rs2282764
rs9878436
rs3093888
rs450962
rs16978028
rs117407747
rs12932179
rs73581419
rs12369950
rs111527438
rs10845387
rs7164950
rs1907702
rs6590343
rs141214782
rs45604339
rs11426156
rs28363070
rs869785
rs139795227
rs6007020
All − MR Egger
All − Inverse variance weighted
−2
−1
0
1
2
MR effect size for
'telomere length || id:ieu−b−4879' on 'IDP_dMRI_TBSS_L2_Tapetum_L || id:ub
